# Progressive Secret Sharing with Adaptive Priority and Perfect Reconstruction

**DOI:** 10.3390/jimaging7040070

**Published:** 2021-04-03

**Authors:** Heri Prasetyo, Chih-Hsien Hsia, Alim Wicaksono Hari Prayuda

**Affiliations:** 1 Department of Informatics, Universitas Sebelas Maret (UNS), Surakarta 57126, Indonesia; wicayudha.wy@gmail.com; 2 Department of Computer Science and Information Engineering, National Ilan University, Yilan 26047, Taiwan

**Keywords:** adaptive priority weight, eXclusive-OR, lossless, odd number, progressive, secret sharing

## Abstract

A new technique for progressive visual secret sharing (PVSS) with adaptive priority weight is proposed in this paper. This approach employs the bitwise and eXclusive-OR (XOR) based approaches for generating a set of shared images from a single secret image. It effectively overcomes the former scheme limitation on dealing with an odd number of stacked or collected shared images in the recovery process. The presented technique works well when the number of stacked shared images is odd or even. As documented in experimental results, the proposed method offers good results over binary, grayscale, and color images with a perfectly reconstructed secret image. In addition, the performance of the proposed method is also supported with theoretical analysis showing its lossless ability to recover the secret image. However, it can be considered as a strong substitutive candidate for implementing a PVSS system.

## 1. Introduction

Recently, several approaches have been devoted to dealing with secure image communication. Transferring a secret image via a communication channel has become an open issue nowadays. Two parties often communicate one to the one another via Internet, cloud computing, communication technology, etc. In this way, a digital image is often transmitted or sent via the communication channels with the security and integrity requirements. A simple means for transferring or exchanging secret information between two or multiple parties is by inserting the secret image into the digital cover image. One can select an appropriate technique for transmitting a secret image. Among of them are the secret sharing technique [1,2,3], the image watermarking technique [4], the multiple secret sharing technique [5,6,7], the progressive secret sharing technique [8,9,10,11,12,13,14,15], the lossless progressive secret image technique [16], etc. These aforementioned methods offer promising performances on in regards to rendering the information of the secret image into the other form (such as a digital image host or shared images).

**Figure 1:**
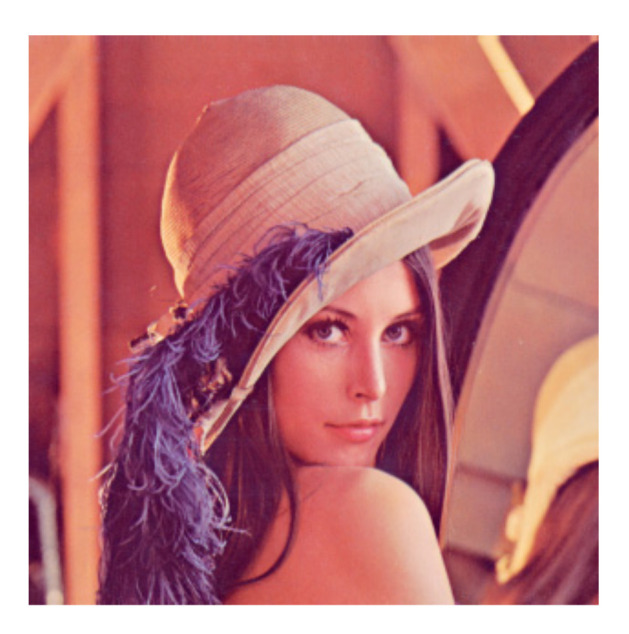
Lena image in color formatting.

**Figure 2:**
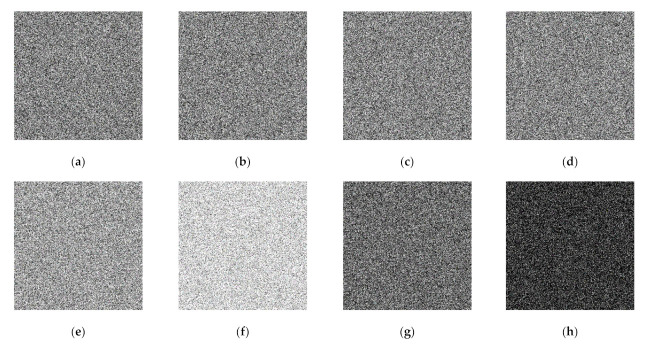
A set of generated shared images: (**a**–**d**)$\left\{S_{1},S_{2},S_{3}S_{4}\right\}$ from the progressive visual secret sharing (PVSS) scheme [16], and (**e**–**h**) $\left\{S_{1},S_{2},S_{3}S_{4}\right\}$ from a PVSS scheme with adaptive priority weight [15].

**Figure 3:**
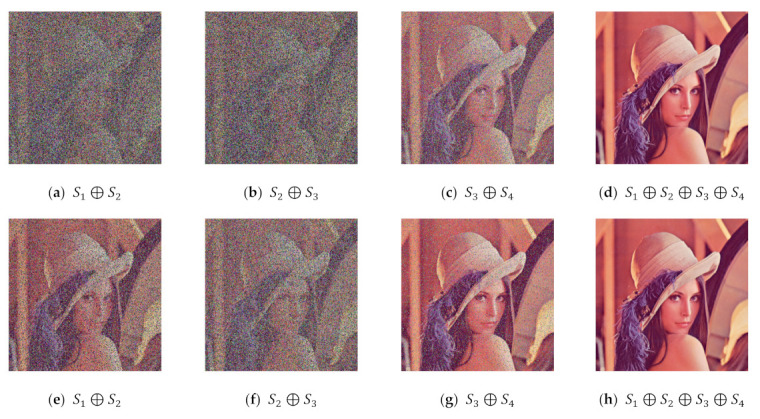
The quality of the reconstructed secret images obtained from: (**a**–**d**) the PVSS scheme [16] and (**e**–**h**) the PVSS method with adaptive priority weight [15].

The secret sharing method aims to convert a meaningful secret image into a non-friendly appearance before transmission to the other parties. The noise-like form can be selected to hide the content of secret image such that an unauthorized malicious attacker cannot recognize the important information contained in the secret image. In recent years, a lot of methods have been developed in the field of secret sharing. The most well-known secret image methods are multiple secret sharing [5,6,7], progressive secret sharing [8,9,10,11,12,13,14,15], lossless progressive secret sharing [16], and more sophisticated secret sharing techniques. The multiple secret sharing method [5,6,7] changes a set of secret images into multiple images or a set of shared images, whereas the progressive secret sharing method simply converts a single secret image into a set of shared images. In the multiple secret sharing method, all shared images are required to reconstruct the secret image. If only a partial subset of shared images is involved in the recovery process, one obtains nothing. The progressive secret sharing method [8,9,10,11,12,13,14,15] offers different ways to reconstruct the secret image. Either a partial or a full set of shared images may be used to obtain the recovered secret image. A higher number of involved shared images gives a better quality of the recovered secret image, and vice versa. However, the progressive secret sharing method cannot give warranty in the lossless recovered secret image. However, a new technique (namely, lossless progressive secret sharing [16]) is able to recover the secret image with lossless quality. Some modifications have been made to the progressive visual secret sharing (PVSS) method with the adaptive priority weight [15]. We give illustrations of PVSS and PVSS with adaptive priority weight in the following example: Figure 1 displays a Lena image in color format. Figure 2 shows a set of shared images generated from the PVSS method [16] and the PVSS scheme with adaptive priority weight [15]. Figure 3 gives the reconstructed secret image by stacking several images obtained from the PVSS method [16] and the PVSS scheme with adaptive priority weight [15]. The adaptive priority weight offers better results regarding the quality of the reconstructed secret images.

This paper first reviews the former scheme [15] on generating a set of shared images from a secret image and recovering a secret image. The former scheme employs the adaptive priority weight to progressively reconstruct a secret image. The former scheme shows its usability in the PVSS task as reported in [15]. Based on our observation, however, the former scheme suffers from a slight limitation in the secret image recovery process if the number of stacked or collected images is odd. This paper delineates this limitation, along with the theoretical analysis required to prove this issue. Some experiments concerning this limitation are also reported. Thereafter, we develop a new technique for overcoming this problem. This new technique inherits the PVSS with a random grids approach from the former scheme with a simple modification implemented to improve the recovery process when the number of stacked images is odd. This simple modification effectively yields a perfect reconstructed secret image whether the number of stacked images is odd or even. The correctness of the proposed method is also supported by mathematical analysis as well as experimental findings. The proposed PVSS method can be touted as a strong alternative candidate for substitution in place of the former scheme [15].

The former scheme [15] and proposed method exploit the eXclusive-OR (XOR) operation for producing a set of shared images as well as obtaining a recovered secret image. The XOR operation is very simple, with various symmetric properties. These XOR properties are very important for designing the PVSS algorithm. One can produce a set of generated shared images using an XOR operation. Conversely, the XOR operation can be used to recover a secret image without the use of any additional computational techniques. The XOR performs differently with common arithmetic operations. The XOR operator has no negation/inverse operation, while the arithmetic has a negation/inverse operator. For example, the arithmetics addition operator owns the negation operator (i.e., arithmetics subtraction). What follows are some useful XOR properties [7] for the PVSS methods. Herein, we provide examples of each property with both the binary and the decimal number representation.

- **Identity:**

This property indicates that performing XOR over any arbitrary number with zero yields an identical arbitrary number itself. This property is defined as:

(1) A⊕0=A

For example, we have $A={\left(6\right)}_{10}$ (i.e., a number 6 in decimal representation) with the corresponding binary string set as ${\left(110\right)}_{2}$. This property tells us that ${\left(110\right)}_{2}⊕{\left(000\right)}_{2}={\left(110\right)}_{2}$ in binary representation or ${\left(6\right)}_{10}⊕{\left(0\right)}_{10}={\left(6\right)}_{10}$ in decimal format.

- **Performing XOR over “odd number” times:**

If we perform XOR operation on the same arbitrary number over “odd number” times, we will receive this arbitrary number itself. Specifically, this process is denoted as:

(2) $$\underset{n\;\mathrm{is}\;\mathrm{odd}\;\mathrm{number}}{\underbrace{A⊕A⊕\ldots ⊕A}}=A⊕\left(A⊕A\right)=A⊕0=A$$

For example, XOR-ing the decimal number 6 over “odd” times gives $\underset{n\;\mathrm{is}\;\mathrm{odd}\;\mathrm{number}}{\underbrace{6⊕6⊕\ldots ⊕6}}=6⊕\left(6⊕6\right)=6⊕0=6$ or, in binary representation, $\underset{n\;\mathrm{is}\;\mathrm{even}\;\mathrm{number}}{\underbrace{{\left(110\right)}_{2}⊕{\left(110\right)}_{2}⊕\ldots ⊕{\left(110\right)}_{2}}}={\left(110\right)}_{2}⊕\left\{{\left(110\right)}_{2}⊕{\left(110\right)}_{2}\right\}={\left(110\right)}_{2}⊕{\left(000\right)}_{2}={\left(110\right)}_{2}$.

- **Performing XOR over “even number” times:**

In contrast to the previous XOR property, performing an XOR operation on the same arbitrary number over “even number” times yields zero results. This property is shown as follows:

(3) $$\underset{n\;\mathrm{is}\;\mathrm{even}\;\mathrm{number}}{\underbrace{A⊕A⊕\ldots ⊕A}}=A⊕A=0$$

A simple example for this property is in the case $\underset{n\;\mathrm{is}\;\mathrm{even}\;\mathrm{number}}{\underbrace{6⊕6⊕\ldots ⊕6}}=6⊕6=0$ in decimal number representation, or, in binary representation, $\underset{n\;\mathrm{is}\;\mathrm{even}\;\mathrm{number}}{\underbrace{{\left(110\right)}_{2}⊕{\left(110\right)}_{2}⊕\ldots ⊕{\left(110\right)}_{2}}}={\left(110\right)}_{2}⊕{\left(110\right)}_{2}={\left(000\right)}_{2}$.

- **Symmetric Inverse:**

This property is almost similar to the common arithmetic inverse, e.g., an addition operator against the subtraction operator. The XOR has no inverse operator. Yet, the XOR can solely perform symmetric inverse by itself. This property is defined as follows:

(4) If A⊕B=C, Then B⊕C=A.

Suppose that there are two decimal numbers A=6 and B=3, with the corresponding binary strings $A={\left(110\right)}_{2}$ and $B={\left(011\right)}_{2}$, respectively. Thus, we have A⊕B=6⊕3=5 or $A⊕B={\left(110\right)}_{2}⊕{\left(011\right)}_{2}={\left(101\right)}_{2}$. It is implied that C=5 or, in binary representation, $C={\left(110\right)}_{2}$. Conversely, we obtain B⊕C=3⊕5=6 or $B⊕C={\left(011\right)}_{2}⊕{\left(101\right)}_{2}={\left(110\right)}_{2}$. This value is identical to A=6 or ${\left(110\right)}_{2}$, showing that the XOR has a unique symmetric inverse property.

- **Commutative:**

The XOR has a similar property as the common arithmetics (i.e., a commutative property). This property is specified as:

(5) A⊕B=B⊕A

Let A=6 and B=3, or, in binary representation, $A={\left(110\right)}_{2}$ and $B={\left(011\right)}_{2}$. The following computations yield identical results (i.e., $A⊕B={\left(110\right)}_{2}⊕{\left(011\right)}_{2}={\left(101\right)}_{2}$ and $B⊕A={\left(011\right)}_{2}⊕{\left(110\right)}_{2}={\left(101\right)}_{2}$) or, in decimal representation, 6⊕3=3⊕6.

- **Associative:**

This property herein is similar to that of the common arithmetic operation. The XOR operation also offers associative computation as formally defined as follows:

(6) $$A⊕\left(B⊕C\right)=\left(A⊕B\right)⊕C$$

Suppose that we have A=6, B=3, and C=5. This property gives $A⊕\left(B⊕C\right)={\left(000\right)}_{2}$ and $\left(A⊕B\right)⊕C={\left(000\right)}_{2}$ in binary form. This result resembles as in the decimal form $6⊕\left(3⊕5\right)=\left(6⊕3\right)⊕5$. However, the XOR operation has been shown to have an associative property.

The main contribution of this paper is to develop a new technique for PVSS with a lossless ability in the secret image reconstruction process whether the number of stacked shared images is odd or even. The other contribution of this paper is a formal mathematical analysis for showing the limitation and correction of the PVSS method. This work has been motivated with the increased demand for secure image transmissions. This work has also motivated the success rate of lossless PVSS in [16]. The organization of this paper is arranged as follows: Section 2 briefly discusses the former PVSS scheme with adaptive priority [15]. This section also shows the limitation of the former scheme [15] as supported with experimental finding and theoretical analysis. Section 3 describes the proposed PVSS method, with adaptive priority analyzed in detail. The proposed method’s usability is supported with the formal mathematical analysis. Section 4 extensively reports some experimental results. The end of this paper delivers the conclusions and direction for future works.

## 2. Former PVSS Scheme

This section briefly reviews the former PVSS scheme [15] wherein the secret and shared images are in binary format. It presents the step-by-step process of shared image generation and secret image reconstruction. A slight shortcoming of this aforementioned method is also provided in this section along with formal theoretical analysis.

### 2.1. PVSS Scheme for Binary Image

The PVSS scheme in [15] for binary image generation is first presented in this section. This scheme exploits the random grids to perform the secret sharing task. The former method converts a secret image in binary format into a set of shared binary images. The procedure of shared image generation is described as follows: Suppose that I is a binary secret image of size M×N. Each pixel on I is denoted as $I\left(x,y\right)$, for x=1,2,…,M and y=1,2,…,N. Since I is a binary image, then $I\left(x,y\right)$ simply consists of two values (i.e., a black (or 0) and white pixel (or 1)). The former scheme [15] changes I into n shared images $\left\{S_{1},S_{2},\ldots ,S_{n}\right\}$ in pixel-based processing and in a bitwise fashion. The symbol $S_{i}$ is the i-th shared image, for i=1,2,…,n. These sets (i.e., shared images) are further transferred to the receiver or decoder side via communication or transmission channel. Each pixel in the shared image $S_{i}$ is denoted as $S_{i}\left(x,y\right)$, for x=1,2,…,M and y=1,2,…,N. Herein, the size of each shared image is identical to that of the size of the secret image (i.e., M×N).

**Figure 4:**
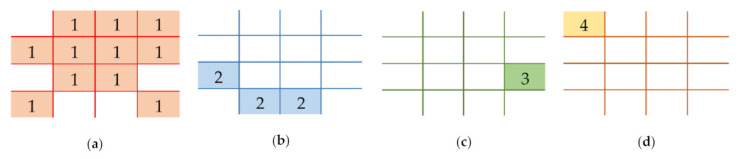
Examples of spatial pixel locations with various adaptive priority weights $\left\{w_{1}=0.5,w_{2}=0.3,w_{3}=0.1,w_{4}=0.1\right\}$: (**a**) $l_{1}$, (**b**) $l_{2}$, (**c**) $l_{3}$, and (**d**) $l_{4}$.

In order to convert a secret image into a set of shared images, the former scheme [15] requires a set of spatial pixel locations ($l_{j}$) for j=1,2,…,n. These locations are specified by their adaptive priority weight ($w_{j})$ for j=1,2,…,n. The adaptive priority weight can be predetermined based on user preference. Figure 4 exhibits some examples of spatial pixel location ($l_{j}$) for j=1,2,…,4, over various adaptive priority weights $\left\{w_{1}=0.5,w_{2}=0.3,w_{3}=0.1,w_{4}=0.1\right\}$. In this figure, the pixel location $l_{1}$ owns around 50% of the occupied pixels in order to generate a shared image. The $l_{1}$ has a higher adaptive weight compared to the other spatial pixel locations. In this example, the additional pixels in $l_{1}$ are caused by the rounding operator in the adaptive priority weight determining the spatial pixel location. Setting a higher priority weight $w_{j}$ indicates more pixel locations $l_{j}$ arranged in the j-th shared image, and vice versa. Thus, the recovered secret image becomes quickly or easily recognized by human vision in the reconstruction process. In addition, the correct pixel of the recovered secret image will be rapidly obtained by utilizing a higher priority weight.

The generation of shared images is formally defined as follows: For each pixel in the secret image (i.e., $I\left(x,y\right)$) we first must determine two selected indices of shared images for the purpose of encoding. The first and second indices of the selected shared images are denoted as $r_{1}$ and $r_{2}$. The value of $r_{1}$ is determined based on the information of the pixel location $l_{j}$, whereas $r_{2}$ is simply computed as $r_{2}\overset{\;}{\leftarrow }\mathrm{mod}\left\{r_{1},n\right\}+1$. The symbols $\mathrm{mod}\left\{\cdot ,\cdot \right\}$ and $\overset{\;}{\leftarrow }$ indicate the arithmetic modulus operator and the assignment operator, respectively. A pixel in a spatial position $\left(x,y\right)$ in the first selected shared image is assigned the following value:

(7) $$S_{r_{1}}\left(x,y\right)\overset{\;}{\leftarrow }U_{I}\left(0,1\right),$$

where $S_{r_{1}}\left(x,y\right)$ represents the pixel in the spatial position $\left(x,y\right)$ over the $r_{1}$-th shared image. The computation in Equation (7) requires a random number generator. Herein, $U_{I}\left(0,1\right)$ denotes the uniformly random number generator producing the integer in range $\left[0,1\right]$. A different strategy is then applied to determine the pixel value of the second selected shared image $S_{r_{2}}\left(x,y\right)$. The pixel in the secret image specifies the value of $S_{r_{2}}\left(x,y\right)$. If the processed secret image is a black pixel (i.e., $I\left(x,y\right)=0$), then the pixel value in $S_{r_{2}}\left(x,y\right)$ is assigned as:

(8) $$S_{r_{2}}\left(x,y\right)\overset{\;}{\leftarrow }S_{r_{1}}\left(x,y\right).$$

If the secret image is a white pixel (i.e., $I\left(x,y\right)=1$), the former scheme provides the pixel $S_{r_{2}}\left(x,y\right)$ as follows:

(9) $$S_{r_{2}}\left(x,y\right)\overset{\;}{\leftarrow }∼S_{r_{1}}\left(x,y\right),$$

where ∼ is the bitwise NOT operator. Subsequently, the pixel values of all shared images excluding $S_{r_{1}}$ and $S_{r_{2}}$ (i.e., all $S_{i}$ for 1≤i≤n and $i\neq r_{1},r_{2}$) are set as $S_{r_{2}}\left(x,y\right)$:

(10) $$S_{i}\left(x,y\right)\overset{\;}{\leftarrow }S_{r_{2}}\left(x,y\right).$$

This process is conducted for all pixels in the secret image I. A set of shared images $\left\{S_{1},S_{2},\ldots ,S_{n}\right\}$ is created at the end of this process. The shared image is in binary format if the binary secret image is fed into the former scheme [15]. Algorithm 1 explains the shared image generation process in detail.
**Algorithm 1:** Former Scheme [15].**Input:** Secret image in binary format, I, of size M×NNumber of shared images, n
**Output:** A set of generated shared images, $\left\{S_{1},S_{2},\ldots ,S_{n}\right\}$, each of size M×N
**Step 1:** Based on priority weight $w_{j}$, determine the location set $l_{j}$, for j=1,2,…,n.**Step 2:** For Each Pixel $\left(x,y\right)$. Based on information $l_{j}$, select two shared images $r_{1}$ and $r_{2}$. Do**Step 3:**$S_{r_{1}}\left(x,y\right)\overset{\;}{\leftarrow }U_{I}\left(0,1\right)$**Step 4:***If*$I\left(x,y\right)=0$, then $S_{r_{2}}\left(x,y\right)\overset{\;}{\leftarrow }S_{r_{1}}\left(x,y\right)$**Step 5: *Else***$S_{r_{2}}\left(x,y\right)\overset{\;}{\leftarrow }∼S_{r_{1}}\left(x,y\right)$**Step 6: *For Each*** other shared images, $S_{i}$, with condition 1≤i≤n and $i\neq r_{1},r_{2}$
*Do*
**Step 7:**$S_{i}\left(x,y\right)\overset{\;}{\leftarrow }S_{r_{2}}\left(x,y\right)$**Step 8:** Obtain n generated shared images, $\left\{S_{1},S_{2},\ldots ,S_{n}\right\}$


In normal situations, all shared images are transmitted to the decoder or receiver side. However, the receiver often collects a partial set of shared images to reconstruct the secret image. Let $\left\{S_{t_{1}},S_{t_{2}},\ldots ,S_{t_{T}}\right\}$ be a partial set of collected shared images in the receiver side, where $t_{1},t_{2},\ldots ,t_{T}$ denotes the index of the received shared image and T≤n. The reconstruction process of the secret image can be easily performed by stacking a partial set of collected shared images with the bitwise XOR-based operation. This process is descibed as follows:

(11) $$\tilde{I}=S_{t_{1}}⊕S_{t_{2}}⊕\ldots ⊕S_{t_{T}},$$

where $\tilde{I}$ is a recovered secret image. The quality can be improved if the PVSS scheme [15] involves more stacked shared images in the secret image reconstruction process. Hopefully, the quality of the recovered secret image would be as similar as possible to that of the original secret image by stacking all shared images with the XOR operator.

### 2.2. Limitation of PVSS Scheme

As reported in literature [15], the former PVSS scheme offers a promising result in the shared image generation and secret image reconstruction processes. The former scheme yields a correct reconstructed secret image if and only if the number of stacked shared images is even. However, it is little regrettable that the former PVSS scheme cannot restore the secret image if the number of stacked shared images is odd. The following theorem explains this limitation.

**Theorem** **1.** *The former PVSS scheme [15] yields perfect or partial reconstruction if and only if the number of stacked shared images is even.*

**Proof.**  *Let*$\left\{S_{t_{1}},S_{t_{2}},\ldots ,S_{t_{T}}\right\}$*be a set of collected or received shared images involved to reconstruct a secret image. In this proof, we investigate the quality of*$\tilde{I}$. *The reconstruction process is performed with the XOR operation in a bitwise-based manner over all collected shared images. The reconstruction process is defined as:*

$$\tilde{I}=S_{t_{1}}⊕S_{t_{2}}⊕\ldots ⊕S_{t_{T}},$$

*If the receiver module collects all shared images, it is implied that*T=n. *The recovered secret image*$\tilde{I}$*can be obtained as:*

(12) $$\tilde{I}=S_{1}⊕S_{2}⊕\ldots ⊕S_{n}.$$

*There are two selected shared images*$(S_{r_{1}}$*and*$S_{r_{2}}$) *in the shared image generation. The other shared images have the value of*$S_{r_{2}}$*(i.e.,*$S_{i}\overset{\;}{\leftarrow }S_{r_{2}}$*for*1≤i≤n*and*$i\neq r_{1},r_{2}$). *For sake of simplicity, we remove the pixel position*$\left(x,y\right)$*for all proofs in this paper. Thus, the form in Equation (12) can be alternatively computed as:*

$$\tilde{I}=S_{r_{2}}⊕S_{r_{2}}⊕\ldots ⊕S_{r_{1}}⊕\ldots ⊕S_{r_{2}}⊕\ldots ⊕S_{r_{2}}$$

*Arranging the* $S_{r_{1}}$ *and* $S_{r_{2}}$ *in an orderly fashion, we gain the following form:*

$$\tilde{I}=S_{r_{1}}⊕S_{r_{2}}⊕\underset{n-2}{\underbrace{S_{r_{2}}⊕S_{r_{2}}⊕\ldots ⊕S_{r_{2}}}}$$

*The value of* n−2 *is an even number if* n *is an even number. This implies the computation of* $\tilde{I}$ *as follows:*

(13) $$\tilde{I}=S_{r_{1}}⊕S_{r_{2}}⊕\underset{n-2\;is\;even}{\underbrace{S_{r_{2}}⊕S_{r_{2}}⊕\ldots ⊕S_{r_{2}}}}$$

*The basic property of XOR operation over “even number times” indicates the result*$\underset{n-2\;is\;even}{\underbrace{S_{r_{2}}⊕S_{r_{2}}⊕\ldots ⊕S_{r_{2}}}}=0$. *Thus, the form in Equation (13) can be further simplified as:*

$$\tilde{I}=S_{r_{1}}⊕S_{r_{2}}⊕0$$

*The XOR property with “zero number” produces the following result:*

(14) $$\tilde{I}=S_{r_{1}}⊕S_{r_{2}}$$

*If the observed pixel of the secret image is black (i.e.,*I=0), *then*$S_{r_{1}}=S_{r_{2}}$. *The XOR property for two identical numbers implies the following result:*

(15) $$\tilde{I}=S_{r_{1}}⊕S_{r_{1}}=0$$

*The result in Equation (15) tells that the original and the recovered secret image are identical if the secret image is*0*and*n*is even number. Based on this fact, we can conclude that*$\tilde{I}=I$. *However, one obtains a correct recovered secret image.*

For the situation where n *is an even number and the secret image is a white pixel (i.e.,* I=1), *the second selected shared image is set as* $S_{r_{2}}\overset{\;}{\leftarrow }∼S_{r_{1}}$. *The substitutive computation of* $\tilde{I}$ *in Equation (14) is indicated as follows:*

$$\tilde{I}=S_{r_{1}}⊕∼S_{r_{1}}.$$

*The XOR property on two complementary numbers yields the following result:*

(16) $$\tilde{I}=1$$

*The result in Equation (16) reveals that the qualities of the recovered and the original secret image are identical if*n*is an even number (i.e.,*$\tilde{I}=I$) *while*I*is white. The former scheme [15] yields a correct result if*n*is an even number.*

*If* n *is an odd number, the computation of* $\tilde{I}$ *is given as:*

$$\tilde{I}=S_{r_{1}}⊕S_{r_{2}}⊕\underset{n-2\;is\;odd}{\underbrace{S_{r_{2}}⊕S_{r_{2}}⊕\ldots ⊕S_{r_{2}}}}$$

*The XOR property “odd number times” yields*$\underset{n-2\;is\;odd}{\underbrace{S_{r_{2}}⊕S_{r_{2}}⊕\ldots ⊕S_{r_{2}}}}=S_{r_{2}}$. *The recovered secret image is then:*

$$\tilde{I}=S_{r_{1}}⊕S_{r_{2}}⊕S_{r_{2}}$$

*The XOR operation concerning two identical numbers results in 0. It gives the following result:*

(17) $$\tilde{I}=S_{r_{1}}⊕0=S_{r_{1}}$$

*The recovered image obtained from Equation (17) is actually a random image (i.e.,*$\tilde{I}=S_{r_{1}}$*with*$S_{r_{1}}\overset{\;}{\leftarrow }U_{I}\left(0,1\right)$). *The recovered secret image cannot be correctly produced if*n*is an odd number (i.e.,*$\tilde{I}\neq I$).

*Under the similar deduction for* T<n *(i.e., only a partial set of shared images is involved in the reconstructed process), the image* $\tilde{I}$ *is computed as:*

$$\tilde{I}=S_{t_{1}}⊕S_{t_{2}}⊕\ldots ⊕S_{t_{T}}$$

*Suppose that*$r_{1}<r_{2}\leq T$. *The computation of*$\tilde{I}$*is then given as:*

$$\tilde{I}=S_{t_{1}}⊕S_{t_{2}}⊕\ldots ⊕S_{r_{1}}⊕\ldots ⊕S_{r_{2}}⊕\ldots ⊕S_{t_{T}}$$

*In the former scheme [15], all shared images are simply determined as*$S_{t_{i}}\overset{\;}{\leftarrow }S_{r_{2}}$*for all*$t_{i}$*with*i=1,2,…,T*and*$t_{i}\neq r_{1},r_{2}$. *This condition implies:*

$$\tilde{I}=S_{r_{1}}⊕S_{r_{2}}⊕S_{r_{2}}⊕\ldots ⊕S_{r_{2}}$$

*The XOR properties “even number times” and “odd number times” indicate the following result:*

$$\tilde{I}=\left\{\begin{array}{c} S_{r_{1}}⊕S_{r_{2}}⊕\underset{T-2\;is\;odd}{\underbrace{S_{r_{2}}⊕S_{r_{2}}⊕\ldots ⊕S_{r_{2}}}}=S_{r_{1}}⊕S_{r_{2}}⊕S_{r_{2}}\\ S_{r_{1}}⊕S_{r_{2}}⊕\underset{T-2\;is\;even}{\underbrace{S_{r_{2}}⊕S_{r_{2}}⊕\ldots ⊕S_{r_{2}}}}=S_{r_{1}}⊕S_{r_{2}}⊕0 \end{array}\right.$$

*Then, the image* $\tilde{I}$ *can be finally obtained as follows:*

(18) $$\tilde{I}=\left\{\begin{array}{c} \begin{array}{cc} S_{r_{1}}, & \;If\;T\;is\;odd \end{array}\\ \begin{array}{cc} S_{r_{1}}⊕S_{r_{2}}, & If\;T\;\mathrm{is}\;even \end{array} \end{array}\right.$$

*It is clearly revealed from Equation (18) that the recovered secret image can be perfectly reconstructed if* T *is an even number. Thus, the perfect recovered secret image can be obtained if and only if the number of stacked shared images is even. This concludes the theorem.* ■

## 3. Proposed PVSS Method

This section gives a detailed explanation for the proposed PVSS method. This new method modifies the former scheme [15] for computing a set of generated shared images. This modification is made to achieve the perfect reconstruction result in the recovery process. Similarly to the former scheme [15], the proposed method also incorporates the adaptive priority weight for a progressive recovery process. However, the proposed method and the former scheme employ an identical approach for performing the secret image reconstruction (i.e., stacking several or all shared images using a bitwise XOR approach). This section presents two techniques for the proposed method using the bitwise-based and XOR-based PVSS approaches.

### 3.1. Proposed Bitwise-Based PVSS Method

The proposed bitwise-based PVSS method is discussed in detail in this subsection. It inherits the usability of the former scheme [15] with a slight modification. This simple modification simply solves a minor limitation in [15] present when the number of stacked or collected shared images is odd. The proposed method also utilizes the random grid technique for generating a set of shared images. The proposed bitwise-based PVSS method for computing a set of shared images is formally explained with the following procedure: Suppose I is a binary image of size M×N. The proposed method transforms the secret image I into n shared images. Let $\left\{S_{1},S_{2},\ldots ,S_{n}\right\}$ be a set of generated shared images, and $\left(x,y\right)$ be the spatial position of an image pixel. Similarly to the former scheme [15], the proposed method first determines two selected shared images (denoted as $r_{1}$ and $r_{2})$. The determinations of $r_{1}$ and $r_{2}$ are based on the priority weight $w_{j}$ and location set $l_{j}$. Each pixel $\left(x,y\right)$ in the $r_{1}$-th shared image is set with a uniformly random number as follows:

(19) $$S_{r_{1}}\left(x,y\right)\overset{\;}{\leftarrow }U_{I}\left(0,1\right)$$

for x=1,2,…,M and y=1,2,…,N. Subsequently, each pixel $\left(x,y\right)$ in the $r_{2}$-th shared image is determined by considering the pixel value $I\left(x,y\right)$. If an investigated pixel value is black (i.e., $I\left(x,y\right)=0$), afterward, the value of $S_{r_{2}}\left(x,y\right)$ is assigned as follows:

(20) $$S_{r_{2}}\left(x,y\right)\overset{\;}{\leftarrow }S_{r_{1}}\left(x,y\right)$$

Meanwhile, the value of $S_{r_{2}}\left(x,y\right)$ is simply set with the bit negation of $S_{r_{1}}\left(x,y\right)$ as formulated below:

(21) $$S_{r_{2}}\left(x,y\right)\overset{\;}{\leftarrow }∼S_{r_{1}}\left(x,y\right)$$

In contrast to the former scheme [15], the proposed method solely utilizes the zero value for all pixels in the shared images $S_{i}$ under the constraints 1≤i≤n and $i\neq r_{1},r_{2}$. This strategy is formally defined as:

(22) $$S_{i}\left(x,y\right)\overset{\;}{\leftarrow }0$$

This shared image generation is applied over all pixel values $\left(x,y\right)$, for x=1,2,…,M and y=1,2,…,N. Algorithm 2 summarizes the procedure of the proposed method for computing a set of shared images $\left\{S_{1},S_{2},\ldots ,S_{n}\right\}$.
**Algorithm 2:** Proposed Bitwise-Based PVSS Method.**Input:** Secret image in binary format, I, of size M×NNumber of shared images, n**Output:** A set of generated shared images, $\left\{S_{1},S_{2},\ldots ,S_{n}\right\}$, each of size M×N
**Step 1:** Based on priority weight $w_{j}$, determine the location set $l_{j}$, for j=1,2,…,n.**Step 2:** For Each Pixel $\left(x,y\right)$. Based on information of $l_{j}$, select two shared images $r_{1}$ and $r_{2}$. Do**Step 3:**$S_{r_{1}}\left(x,y\right)\overset{\;}{\leftarrow }U_{I}\left(0,1\right)$**Step 4:** If $I\left(x,y\right)=0$, Then $S_{r_{2}}\left(x,y\right)\overset{\;}{\leftarrow }S_{r_{1}}\left(x,y\right)$
**Step 5:** Else $S_{r_{2}}\left(x,y\right)\overset{\;}{\leftarrow }∼S_{r_{1}}\left(x,y\right)$
**Step 6:** For Each Generated shared images, $S_{i}$, with the condition 1≤i≤n and $i\neq r_{1},r_{2}$ Do **Step 7:**$S_{i}\left(x,y\right)\overset{\;}{\leftarrow }0$**Step 8:** Obtain n generated shared images, $\left\{S_{1},S_{2},\ldots ,S_{n}\right\}$


Similar to [15], the proposed method collects a partial or full set of generated shared images in order to recover the secret image. Herein, the proposed method also performs an XOR operation over these collected shared images. This process is defined as follows:

(23) $$\tilde{I}=S_{t_{1}}⊕S_{t_{2}}⊕\ldots ⊕S_{t_{T}}$$

where $\tilde{I}$ denotes the recovered secret image and $t_{T}$ is the total number of collected shared images on the receiver side. The proposed bitwise-based PVSS method is quite simple, yet it effectively solves the lossless problem in [15]. The following analysis supports the proposed method performance theoretically.

**Theorem** **2.** *The proposed bitwise-based PVSS method yields a perfectly reconstructed secret image by stacking a partial or full set of generated shared images.*

**Proof.**  *We begin this proof with the quality of the recovered secret image produced by the proposed bitwise-based PVSS method. The XOR-ed process over a partial or full set of generated shared images is denoted as:*

$$\tilde{I}=S_{t_{1}}⊕S_{t_{2}}⊕\ldots ⊕S_{t_{T}}$$

*We first investigate the proposed method performance when one involves all shared images in the recovery process. In this occasion, it similarly performs a recovery process under a condition*T=n. *However, the computation of*$\tilde{I}$*can be performed as follows:*

(24) $$\tilde{I}=S_{1}⊕S_{2}⊕\ldots ⊕S_{n}$$

*We know that*$1\leq r_{1},r_{2}\leq n$*and*$S_{i}\overset{\;}{\leftarrow }0$*for all*1≤i≤n*and*$i\neq r_{1},r_{2}$. *This implies that Equation (24) can be recalculated when considering*n*as an even or odd number as follows:*

(25) $$\tilde{I}=S_{r_{1}}⊕S_{r_{2}}⊕0⊕\ldots ⊕0,\;\tilde{I}=S_{r_{1}}⊕S_{r_{2}}⊕\underset{n-2\;is\;odd/even\;number}{\underbrace{0⊕0⊕\ldots ⊕0⊕0}}$$

*Performing XOR on any arbitrary number with zero is equivalent to the arbitrary number itself. Thus, the Equation (25) can be simplified as:*

(26) $$\tilde{I}=S_{r_{1}}⊕S_{r_{2}}$$

*Stacking all shared images actually resembles the process of performing an XOR operation between*$S_{r_{1}}$ and $S_{r_{2}}$. *In addition, the proposed method gives various values for* $S_{r_{2}}$, *depending on the value of* I. If I=1*, then the value of* $S_{r_{2}}$ *is identically set with the value of* $S_{r_{1}}$. *While* I=0*, the value of* $S_{r_{2}}$ *is set in bitwise negation of* $S_{r_{1}}$ *(i.e.,* $S_{r_{2}}\overset{\;}{\leftarrow }S_{r_{1}}$). *This condition gives* $\tilde{I}$ *as follows:*

$$\tilde{I}=\left\{\begin{array}{c} \begin{array}{cc} S_{r_{1}}⊕S_{r_{1}}, & If\;I=0 \end{array}\\ \begin{array}{cc} S_{r_{1}}⊕∼S_{r_{1}}, & If\;I=1 \end{array} \end{array}\right.$$

*The following result is obtained based on the XOR property:*

(27) $$\tilde{I}=\left\{\begin{array}{c} \begin{array}{cc} 0, & If\;I=0 \end{array}\\ \begin{array}{cc} 1, & If\;I=1 \end{array} \end{array}\right.$$

*The last form indicates an important result (i.e.,* $\tilde{I}=I$ *). Herein, the recovered and the original secret image are identical. Thus, the proposed method yields a perfectly reconstructed secret image when all shared images are involved in the recovery process.*

*If only a partial set of shared images is involved under condition* T<n *, the recovered secret image* $\tilde{I}$ *can be computed as follows:*

$$\tilde{I}=S_{t_{1}}⊕S_{t_{2}}⊕\ldots ⊕S_{t_{T}}$$

*Suppose that the two selected shared images (* $S_{r_{1}}$ *and* $S_{r_{2}}$ *) are in this partial set under the condition* $r_{1}<r_{2}\leq T$ *. One cannot obtain a perfectly reconstructed secret image if this condition is not satisfied. The computation of* $\tilde{I}$ *is then given as:*

$$\tilde{I}=S_{t_{1}}⊕S_{t_{2}}⊕\ldots ⊕S_{r_{1}}⊕\ldots ⊕S_{r_{2}}⊕\ldots ⊕S_{t_{T}}$$

*Based on the fact that* $S_{i}\overset{\;}{\leftarrow }0$ *for all* 1≤i≤n *and* $i\neq r_{1},r_{2}$ *, one can trivially obtain the following form:*

$$\tilde{I}=S_{r_{1}}⊕S_{r_{2}}⊕S_{t_{1}}⊕\ldots ⊕S_{t_{T}}=S_{r_{1}}⊕S_{r_{2}}⊕\underset{T-2\;is\;odd/even\;number}{\underbrace{0⊕0⊕\ldots ⊕0⊕0}}$$

*The XOR property implies the following result:*

$$\tilde{I}=S_{r_{1}}⊕S_{r_{2}}$$

*The last form indicates that the value of* $\tilde{I}$ *is identical to that of the XOR-ed result between* $S_{r_{1}}$ *and* $S_{r_{2}}$ *. By investigating the value of* I *, we acquire the following conclusion:*

$$\tilde{I}=\left\{\begin{array}{c} \begin{array}{cc} S_{r_{1}}⊕S_{r_{1}}=0, & If\;I=0 \end{array}\\ \begin{array}{cc} S_{r_{1}}⊕∼S_{r_{1}}=1, & If\;I=1 \end{array} \end{array}\right.$$

*In the case of* T<n *, we achieve an important deduction (i.e.,* $\tilde{I}=I$ *) To simplify, the quality of the recovered secret image is identical to that of the original secret image. In addition, a perfect recovered secret image can be yielded if either a partial set or all generated shared images are involved in the reconstruction process. This completes the proof.* ■

### 3.2. Proposed XOR-ed Based PVSS Method

In this approach, the proposed method performs a simple computation involving an XOR operation in order to generate a set of shared images. The proposed method takes an image I of M×N as the secret image to produce the targeted shared images $\left\{S_{1},S_{2},\ldots ,S_{n}\right\}$. Herein, the secret image can be present as binary, grayscale, or color space. For each pixel $\left(x,y\right)$ in the secret image, we perform the following procedure to generate n shared images: We first decide two selected shared images $(r_{1}$ and $r_{2}$). In contrast to the former scheme [15] and the proposed bitwise-based approach, the proposed XOR-based method needs to first generate the following constant:

(28) $$C\overset{\;}{\leftarrow }U_{I}\left(a,b\right)$$

where C is a constant. The symbol $U_{I}\left(a,b\right)$ denotes a uniform random number generator producing an integer in range $\left[a,b\right]$. We utilize $U_{I}\left(0,1\right)$ and $U_{I}\left(0,255\right)$ for the binary image and the 8-bit grayscale image, respectively. A three dimensional image of $U_{I}\left(0,255\right)$ can be used for the 24-bit color image (i.e., generating a random number for three dimensional color spaces). Subsequently, all pixels in two selected shared images ($S_{r_{1}}$ and $S_{r_{2}}$) are determined as follows:

(29) $$S_{r_{1}}\left(x,y\right)\overset{\;}{\leftarrow }I\left(x,y\right)⊕C$$

(30) $$S_{r_{2}}\left(x,y\right)\overset{\;}{\leftarrow }C$$

All pixels in $S_{i}$ are simply set with zero value for 1≤i≤n and $i\neq r_{1},r_{2}$. Alternatively, the pixels are set according to the following process:

(31) $$S_{i}\left(x,y\right)\overset{\;}{\leftarrow }0$$

The proposed XOR-ed PVSS method requires simple steps to compute a set of shared images. This simple approach is also applicable for grayscale and color images. The contents of all shared images are totally different compared to that of the original secret image. In addition, the proposed XOR-based PVSS method is designed to solve a slight problem in the former scheme [15]. Algorithm 3 illustrates the shared image generation using the proposed XOR-based PVSS approach.
**Algorithm 3:** Proposed XOR-ed Based PVSS Method.**Input:** A grayscale or color image as secret, I, of size M×N
Number of shared images, n
**Output:** Full set of generated shared images, $\left\{S_{1},S_{2},\ldots ,S_{n}\right\}$, each of size M×N**Step 1:** Based on priority weight $w_{j}$, determine the location set $l_{j}$, for j=1,2,…,n.**Step 2:** For Each Pixel Position $\left(x,y\right)$**.** Based on the information in $l_{j}$, decide the selected shared images $r_{1}$ and $r_{2}$. Do **Step 3:**$C\overset{\;}{\leftarrow }U_{I}\left(a,b\right)$**Step 4:**$S_{r_{1}}\left(x,y\right)\overset{\;}{\leftarrow }I\left(x,y\right)⊕C$**Step 5:**$S_{r_{2}}\left(x,y\right)\overset{\;}{\leftarrow }C$**Step 6:** For Each other generated shared images, $S_{i}$, under the condition 1≤i≤n and $i\neq r_{1},r_{2}$ Do **Step 7:**$S_{i}\left(x,y\right)\overset{\;}{\leftarrow }0$**Step 8:** Obtain the n generated shared images, $\left\{S_{1},S_{2},\ldots ,S_{n}\right\}$


The proposed method reconstructs the secret image in a similar fashion as compared to the former scheme [15]. Herein, the proposed method simply needs to perform an XOR operation over either a partial set or all generated shared images. The following theorem supports the correctness of the proposed method.

**Theorem** **3.** *The proposed XOR-ed based PVSS method yields a perfectly reconstructed secret image by stacking partial or all generated shared images.*

**Proof.**  *In order to prove this theorem, we first examine the quality of recovered secret image produced by our proposed method. Suppose that all generated shared images are involved in the recovery process of a secret image. This indicates that* T=n *. Thus, the* $\tilde{I}$ *can be produced as follows:*

(32) $$\tilde{I}=S_{1}⊕S_{2}⊕\ldots ⊕S_{n}=S_{r_{1}}⊕S_{r_{2}}$$

*The simplified form in Equation (32) is actually identical to that of Equation (26). The proposed method applies a similar strategy on* $S_{i}$ *(i.e.,* $S_{i}\overset{\;}{\leftarrow }0$ *for* 1≤i≤n *and* $i\neq r_{1},r_{2})$ *. The selected shared images* $S_{r_{1}}$ *and* $S_{r_{2}}$ *are set with the value of* I⊕C *and* C *, respectively. The form in Equation (32) is similar to following computation:*

(33) $$\tilde{I}=\left(I⊕C\right)⊕C\;\tilde{I}=I⊕\left(C⊕C\right)$$

*The XOR property indicates that an XOR operation between two identical scalars yields zero value. Thus, the form in Equation (33) has the following result:*

$$\tilde{I}=I⊕0$$

*The XOR operation between scalar and zero produces the scalar itself. However, one obtains the following result:*

(34) $$\tilde{I}=I$$

*The last form in Equation (34) clearly reveals that the proposed XOR-based PVSS method achieves a lossless result. The qualities of the recovered and the original secret image are identical if all generated shared images are utilized in the recovery process.*

*If only several shared images are involved in the recovery of a secret image (i.e., in the case of* T<n *), the recovered secret image* $\tilde{I}$ *is computed as follows:*

$$\tilde{I}=S_{t_{1}}⊕S_{t_{2}}⊕\ldots ⊕S_{t_{T}}$$

*The condition* $r_{1}<r_{2}\leq n$ *implies the following result:*

(35) $$\tilde{I}=S_{r_{1}}⊕S_{r_{2}}$$

*A similar deduction on Equation (33) can be applied for Equation (35). Thus, we conclude an important result (i.e.,* $\tilde{I}=I)$ *. The proposed XOR-based PVSS method is able to reconstruct a secret image with the lossless condition even if only a partial set of generated shared images is involved in the reconstruction process. The proposed XOR-based PVSS approach yields a perfect recovered secret image whether all or several generated shared images are utilized in the recovery process. This concludes the proof.* ■

## 4. Experimental Results

The performances of the proposed method and the former scheme [15] are extensively reported in this section in terms of dealing with the PVSS tasks. We first explain several image quality assessment metrics to objectively measure the degree of similarity between the original and the recovered secret image. Subsequently, the performances of the proposed method are compared under visual investigation and objective measurement over binary, grayscale, and color images. These assessments are conducted to further investigate the proposed method’s usability and superiority. The comparisons in terms of algorithm aspects between the proposed method and competing schemes are summarized at the end of this section.

### 4.1. Performance Evaluation

We first evaluated the performance under the subjective and objective assessments. For the subjective image quality assessment, the quality of the recovered secret image was simply inspected and judged based on a visual observation. Herein, we visually compared the similarities between the original and the recovered secret image under the perception of human vision, whereas the objective image quality assessment utilizes several metrics to calculate the degree of similarity between the original and the recovered secret image. These metrics are referred to as image contrast α, bit error rate β, peak signal-to-noise ratio (PSNR), structural similarity index metric (SSIM), and mean absolute error (MAE). All of these objective metrics are formally defined as follows:

(36) $$\alpha =\frac{T\left(\tilde{I}\left[I\left(1\right)\right]\right)-T\left(\tilde{I}\left[I\left(0\right)\right]\right)}{1+T\left(\tilde{I}\left[I\left(0\right)\right]\right)}$$

(37) $$\beta =\frac{\sum_{x=1}^{M}\sum_{y=1}^{N}I\left(x,y\right)⊕\tilde{I}\left(x,y\right)}{MN}$$

(38) $$PSNR(I,\tilde{I})\;=20\log_{10}\frac{255}{\frac{1}{MN}\sum_{i=1}^{M}\sum_{j=1}^{N}{\left[I\left(i,j\right)\;-\;\tilde{I}\left(i,j\right)\right]}^{2}}$$

(39) $$SSIM\left(I,\tilde{I}\right)=\frac{\left(2\mu_{I}\mu_{\tilde{I}}+c_{1}\right)\left(2\sigma_{I\tilde{I}}+c_{2}\right)}{\left(\mu_{I}^{2}+\mu_{\tilde{I}}^{2}+c_{1}\right)\left(\sigma_{I}^{2}+\sigma_{\tilde{I}}^{2}+c_{2}\right)}$$

(40) $$MAE\left(I,\tilde{I}\right)=\frac{1}{MN}\overset{M}{\underset{i=1}{\sum }}\overset{N}{\underset{j=1}{\sum }}\left|I\left(i,j\right)-\tilde{I}\left(i,j\right)\right|$$

where I is the secret image, and $\tilde{I}$ is the recovered secret image. These two images are of the same size (i.e., M×N).

In the SSIM computation, the symbols $\mu_{I}$ and $\mu_{\tilde{I}}$ are the mean values of I and $\tilde{I}$, respectively. However, the standard deviations of I and $\tilde{I}$ are denoted as $\sigma_{I}$ and $\sigma_{\tilde{I}}$, respectively. Meanwhile, the covariance between I and $\tilde{I}$ is denoted as $\sigma_{I\tilde{I}}$. The $c_{1}$ and $c_{2}$ are two predetermined constants. In the case of a binary image, the symbols $T\left(\tilde{I}\left[I\left(1\right)\right]\right)$ and $T\left(\tilde{I}\left[I\left(0\right)\right]\right)$ denote the average light transmission [8] of the recovered secret images over a white pixel (1) and a black pixel (0), respectively. In our subsequent experiment, a better performance is indicated by higher scores of α, PSNR, and SSIM, and vice versa. On the other hand, a better performance is also implied by lower values of β and MAE, and vice versa.

### 4.2. Visual Evaluation on Binary Image

**Figure 5:**
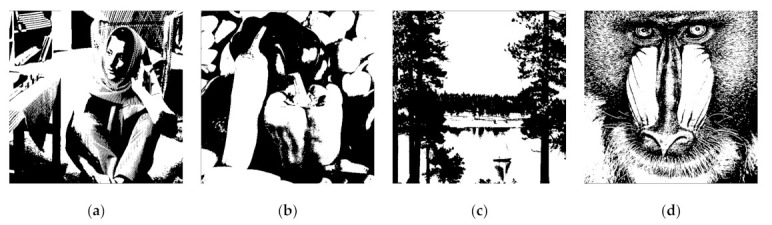
Four secret images in a binary format for experiment (**a**) $I_{1}$, (**b**) $I_{2}$, (**c**) $I_{3}$, and (**d**) $I_{4}$.

**Figure 6:**
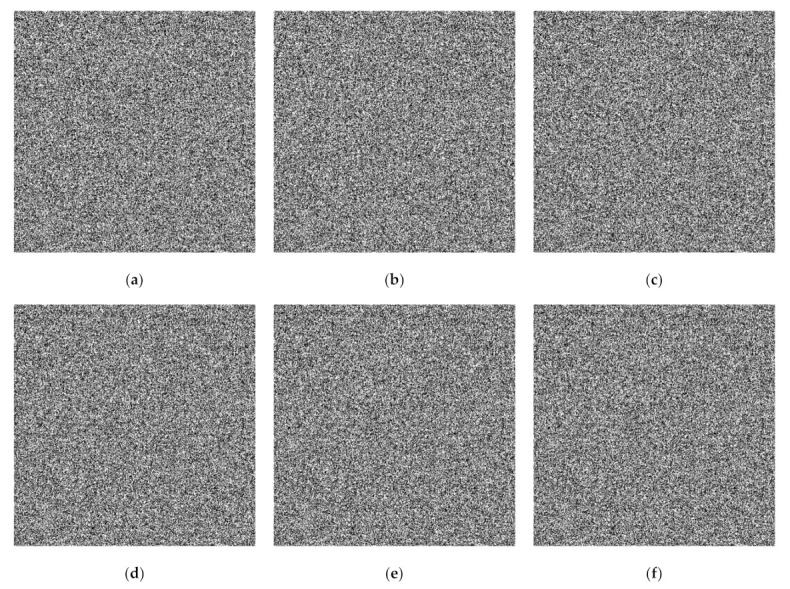
Generated binary shared images using the proposed bitwise-based PVSS method for n=6: (**a**–**f**) $\left\{S_{1},S_{2},\ldots ,S_{6}\right\}$.

The visual investigation between the proposed method and the former scheme [15] in terms of a binary image is reported in this subsection. We examined the performances of the proposed method and the former scheme [15] under a set of binary images as displayed in Figure 5. In this experiment, we simply set the adaptive priority weights $w_{j}$ as $\left\{0.4,\;0.3,\;0.15,\;0.1,\;0.025,\;0.025\right\}$ and $\left\{0.4,\;0.3,\;0.2,\;0.05,\;0.05\right\}$ for n=6 and n=5, respectively. Figure 6 exhibits a set of generated shared images for n=6 with the proposed bitwise-based PVSS method. Setting a higher value for adaptive priority weight implies a brighter shared image compared to that obtained by setting a lower value of $w_{j}$. In addition, the contents of all generated shared images are in a noise-like appearance, meaning that each image cannot easily be distinguished. This clearly reveals that the proposed method satisfies the PVSS constraint (i.e., that the content of the generated shared images cannot be recognized by an unauthorized party).

**Figure 7:**
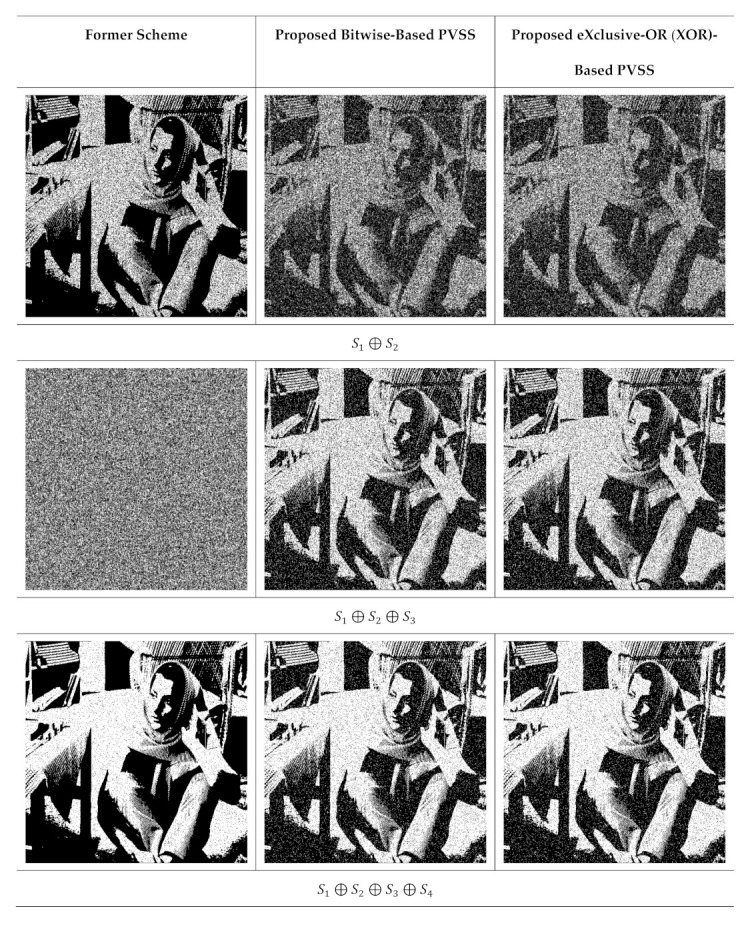

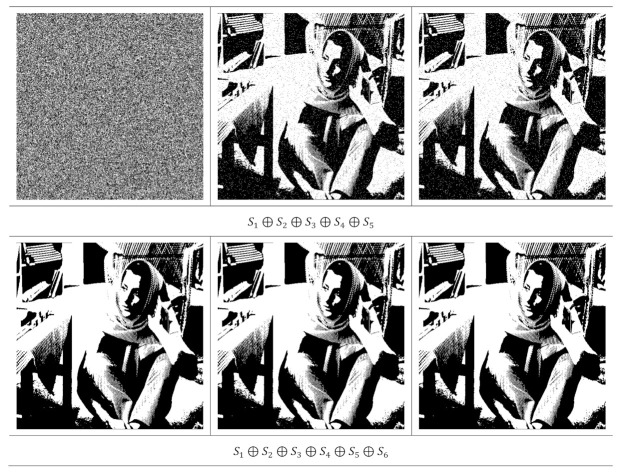
The results of stacking several shared images with t=2,3,…,6, by setting n=6. The first colum is from the former scheme [15], while the second and third columns are from the proposed method.

**Figure 8:**
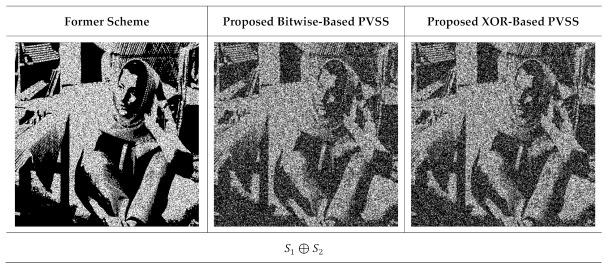

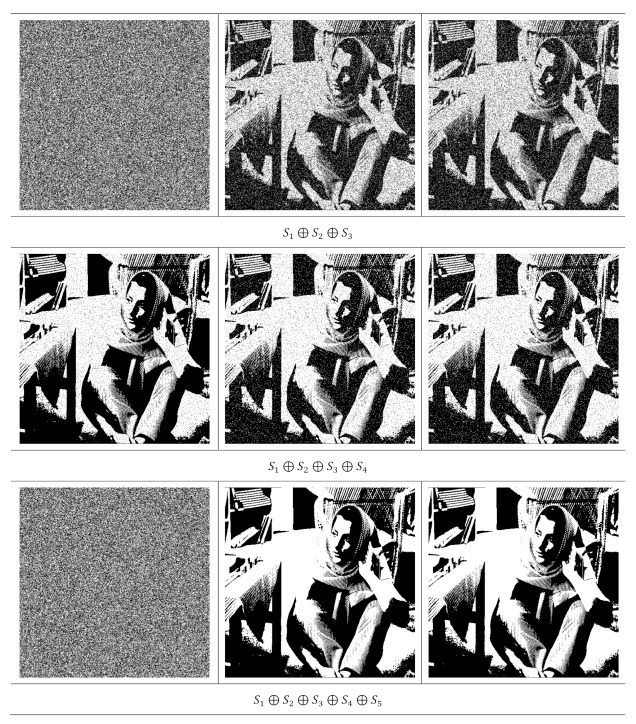
Stacking several shared images t=2,3,…,5, by setting n=5. The first column is from the former scheme [15], while the second and third columns are from the proposed method.

Subsequently, we verified the quality of the recovered secret image. We select Barbara from Figure 5a as a binary secret image. We investigated and compared the performances of the proposed method and former scheme using visual inspection of the recovered secret image. Figure 7 displays the recovery process of the Barbara secret image for n=5, while Figure 8 displays the recovery process for n=6. These two figures demonstrated the superiority of the proposed method compared to that of [15]. The proposed method was able to produce the recovered secret image when the number of stacked shared image T is odd. However, one cannot reconstruct the secret image using the former scheme [15] if T is an odd number. In addition, the recovered secret image produced by the proposed method is lossless if all shared images are stacked using an XOR operation. This experiment indicates that the proposed method offers a promising result in the PVSS task.

### 4.3. Visual Investigation on Grayscale Image

**Figure 9:**
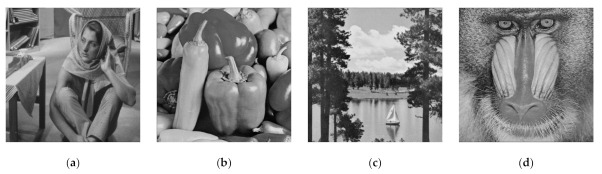
Four grayscale images as secret for experiment: (**a**–**d**) $\left\{I_{1},I_{2},I_{3},I_{4}\right\}$.

**Figure 10:**
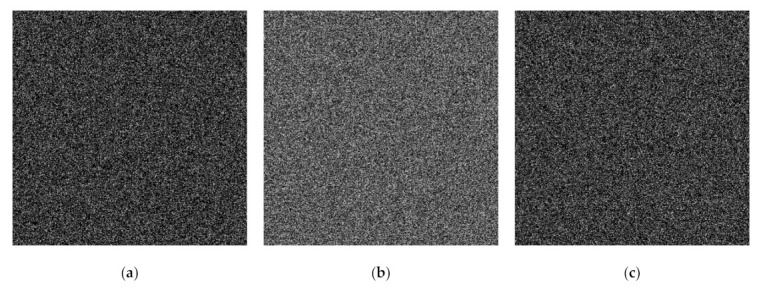

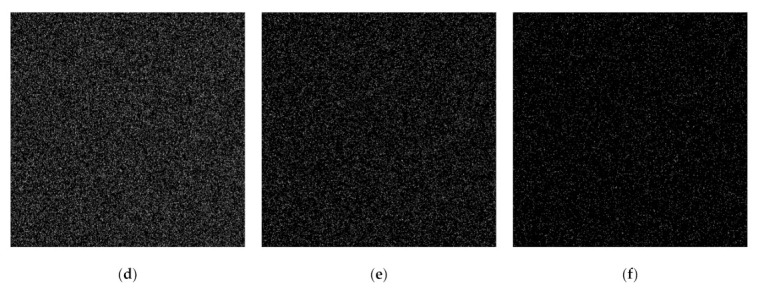
Generated shared images using the proposed eXclusive-OR (XOR)-based PVSS method: (**a**–**f**) $\left\{S_{1},S_{2},\ldots ,S_{6}\right\}$, by setting n=6.

We subsequently considered the performance of the proposed method and the former scheme [15] under visual investigation. In this experiment, we examined the performances of four secret images in grayscale, as shown in Figure 9. The adaptive priority weights were identically set to those used in the binary image case. In the shared image generation, we simply employed n=5 and n=6. Figure 10 displays a set of shared images for n=6 when the Barbara grayscale image is selected as a secret image. As depicted in this figure, the content of the generated shared images cannot be perceived and understood by human vision. This means that the shared images are effectively produced by the proposed method.

**Figure 11:**
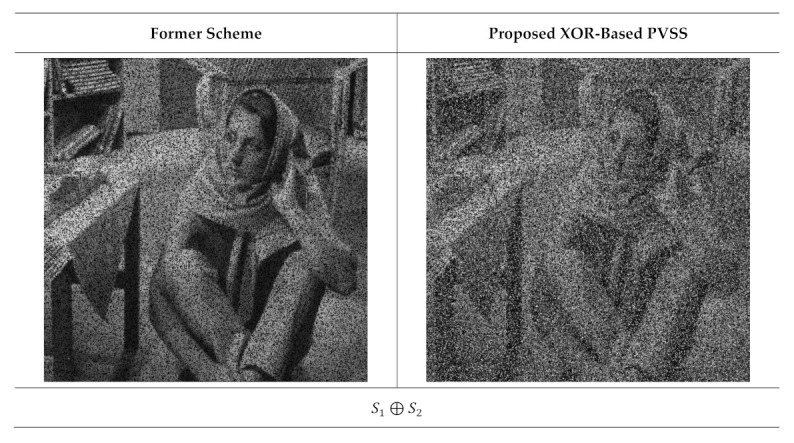

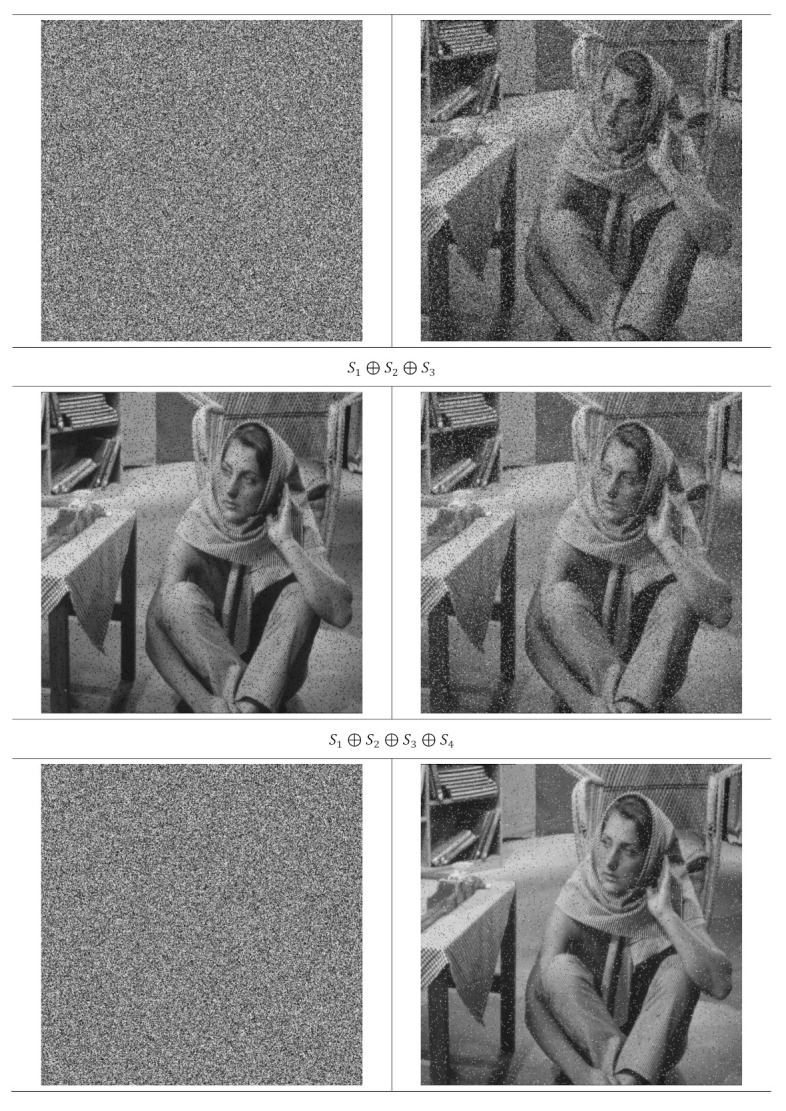

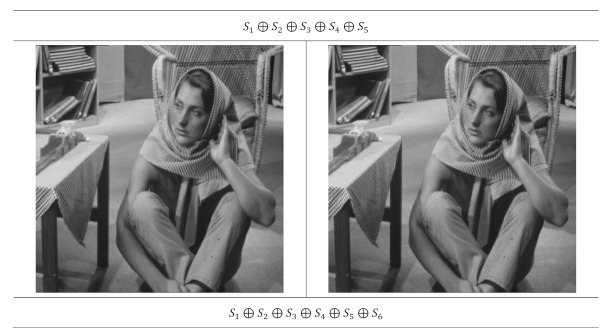
Stacking several shared images with t=2,3,…,6 and n=6. The left and right columns are from the former scheme [15] and the proposed method, respectively.

**Figure 12:**
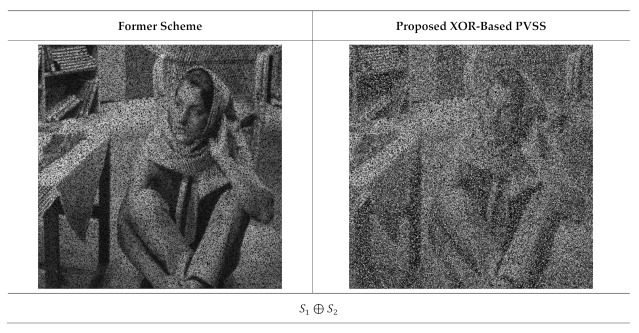

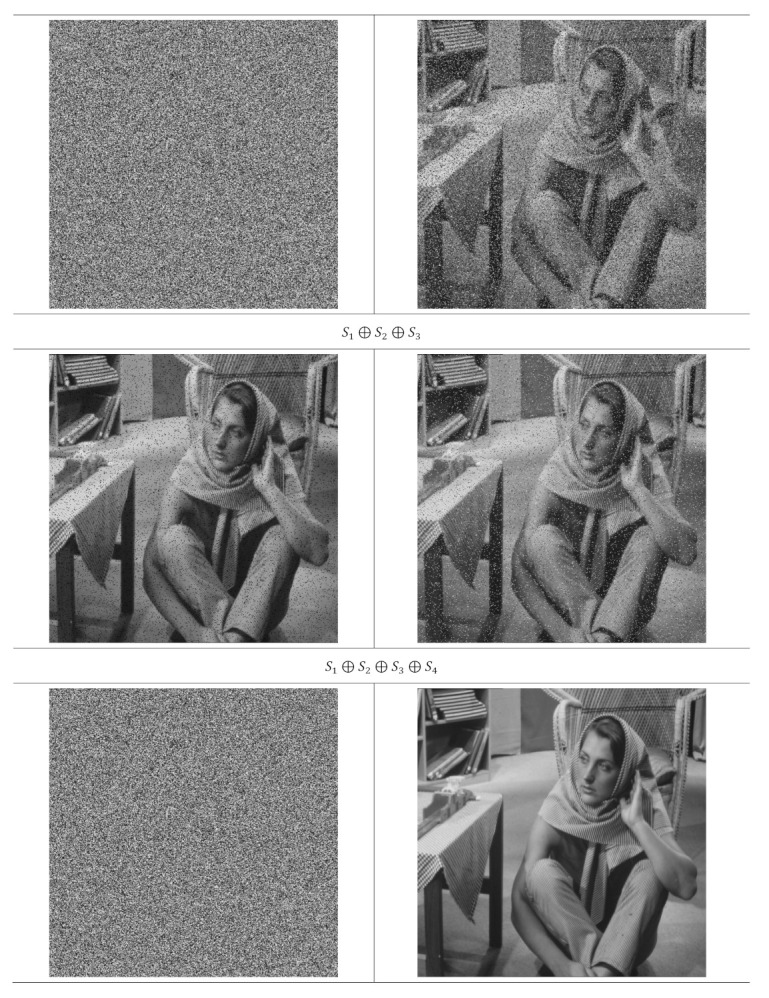

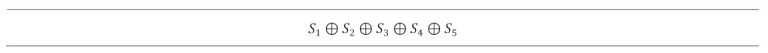
Reconstructed secret images when several shared images are stacked, with t=2,3,…,5 and n=5. The left and right columns are from the former scheme [15] and the proposed method, respectively.

The qualities of the recovered secret image were further inspected under visual investigation. Herein, the recovered secret image produced by stacking several shared images is shown in Figure 11 and Figure 12 for n=6 and n=5, respectively. As shown in these two figures, the quality of the recovered secret image is increased if more shared images are involved in the reconstruction process. However, the former scheme [15] produces an incorrectly recovered secret image if the number of stacked shared images is odd (i.e., the content of the recovered secret image cannot be correctly reconstructed after the stacking process). Conversely, the proposed XOR-based method works well, indicating its superiority compared to that of the former scheme [15].

### 4.4. Visual Assessment of Color Image

**Figure 13:**
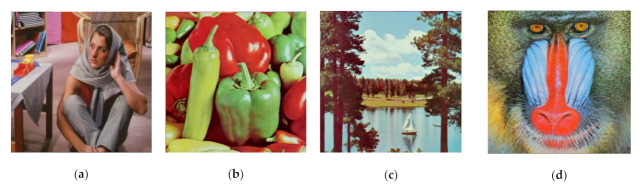
A set of color images used as secret images in the experiment, denoted as: (**a**) $I_{1}$, (**b**) $I_{2}$, (**c**) $I_{3}$, and (**d**) $I_{4}$

**Figure 14:**
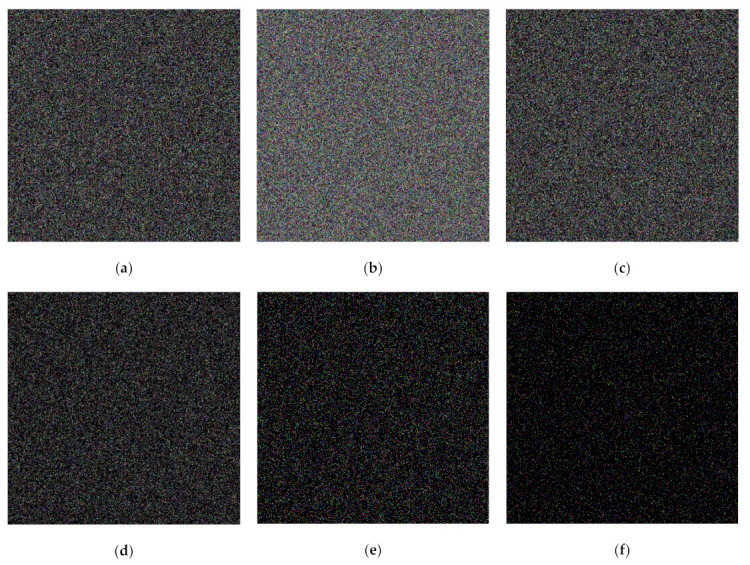
Shared images obtained from the secret image in the color format using the proposed XOR-based PVSS method: (**a**–**f**) $\left\{S_{1},S_{2},\ldots ,S_{6}\right\}$.

This subsection compares the performances of the former scheme [15] and the proposed method under the visual inspection on color image. Four color images (as shown in Figure 13) were used for experimentation. Herein, the number of shared images is set as n=5 and n=6. We applied an identical adaptive priority weight, as used in the binary image case. Figure 14 displays a set of shared images in color format, while the color image in Figure 13a was chosen as a secret image. Human vision cannot recognize the object or image content from all shared images as delivered in Figure 14. Thus, it can be concluded that the proposed method effectively produces a set of shared images in color format.

**Figure 15:**
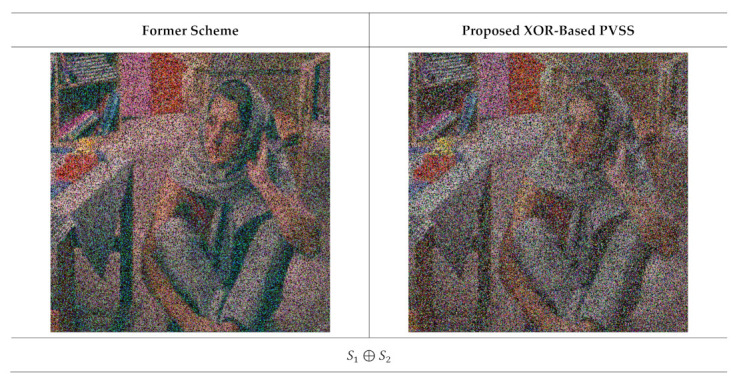

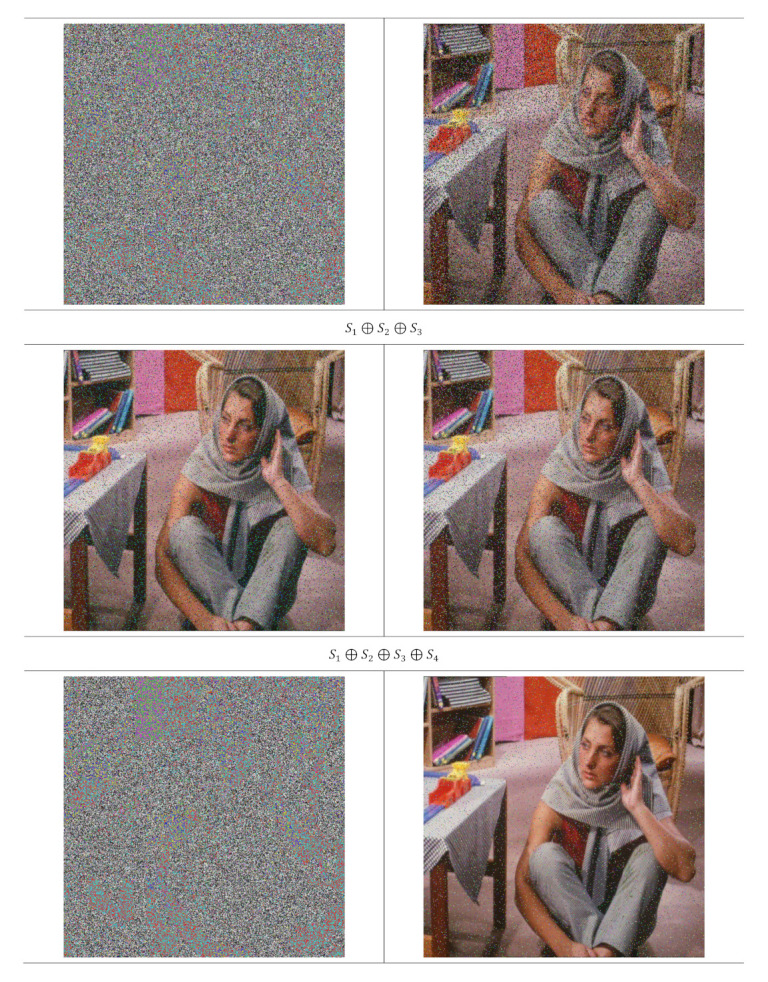

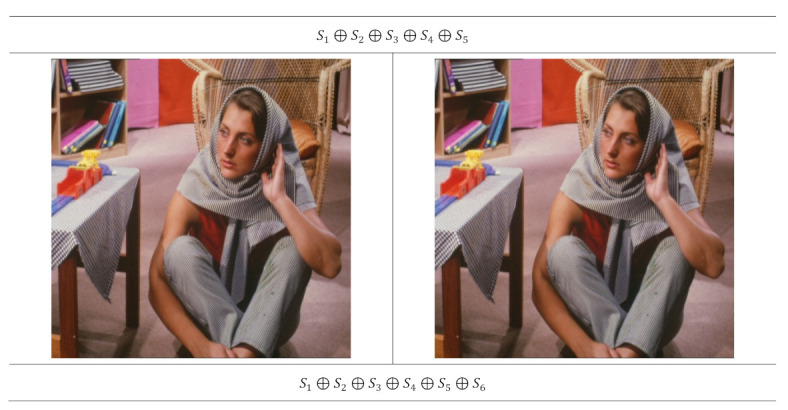
The results of stacking several shared images with t=2,3,…,6 and n=6. The left and right columns are from the former scheme [15] and the proposed method, respectively.

**Figure 16:**
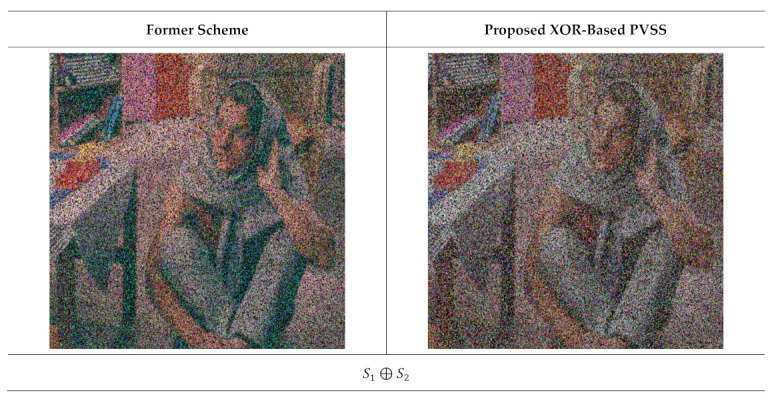

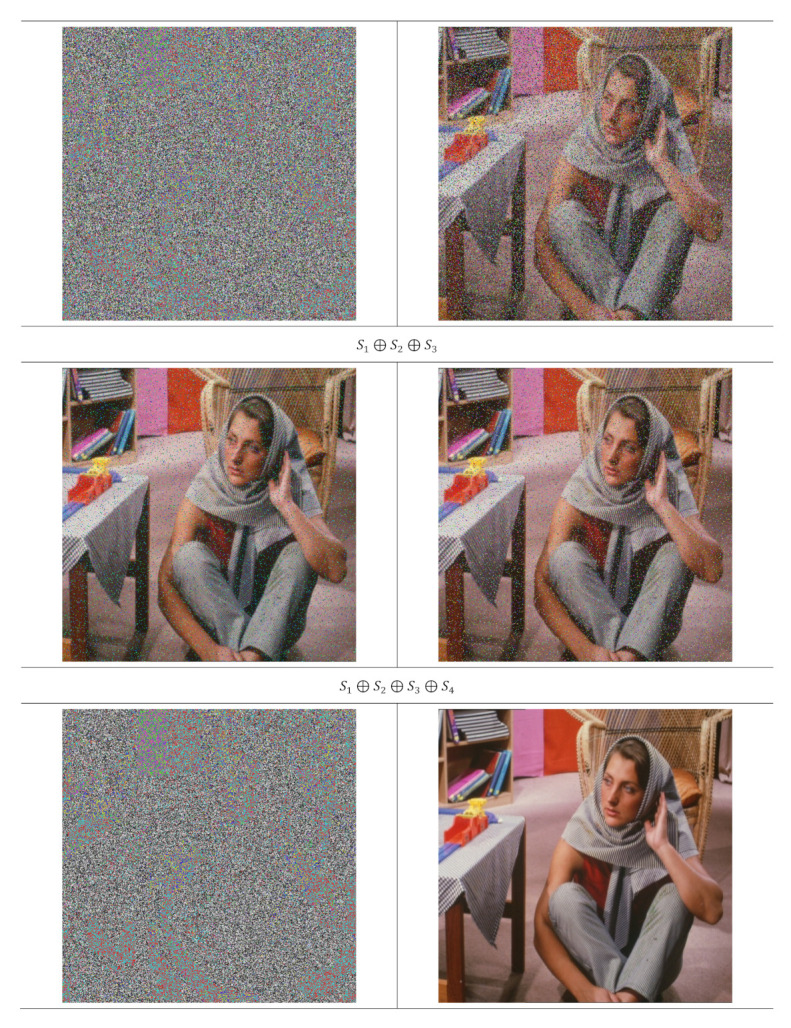

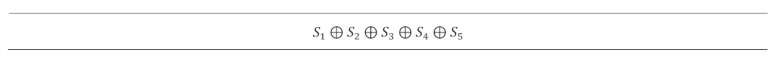
The results of stacking several shared images t=2,3,…,5 and n=5. The left and right columns are the recovered secret image from the former scheme [15] and the proposed method, respectively.

Subsequently, we observed the quality of the recovered secret image after stacking several shared images using an XOR operation. In this experiment, we reconstructed the secret image by stacking two shared images until reaching n shared images. Figure 15 displays the recovered secret image obtained from the former scheme [15] and the proposed method for n=6, while Figure 16 shows the results for n=5. It can be observed from Figure 15 and Figure 16 that the former scheme [15] and the proposed method satisfy the progressive constraint (i.e., the quality of the recovered secret image is increased if more shared images are involved and stacked with an XOR operation). Similarly to the binary and grayscale image cases, the proposed XOR-based PVSS method produces a good result whether the number of stacked shared images is odd or even, whereas the former scheme [15] cannot correctly yield the recovered secret image if the number of stacked shared images is odd. The proposed XOR-based PVSS overcomes the limitation of [15] with a simple approach. Thus, the proposed method delivers a promising result for binary, grayscale, and color images.

### 4.5. Performance Comparisons in Terms of Objective Image Quality Assessment

**Figure 17:**
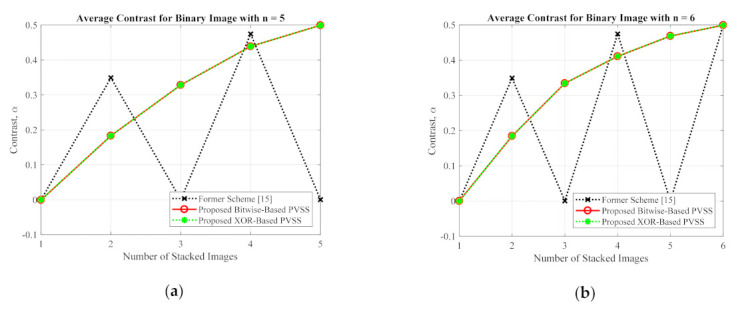
The average image contrast between the proposed method and the former scheme [15] of the binary secret image with (**a**) n=5, and (**b**) n=6.

**Figure 18:**
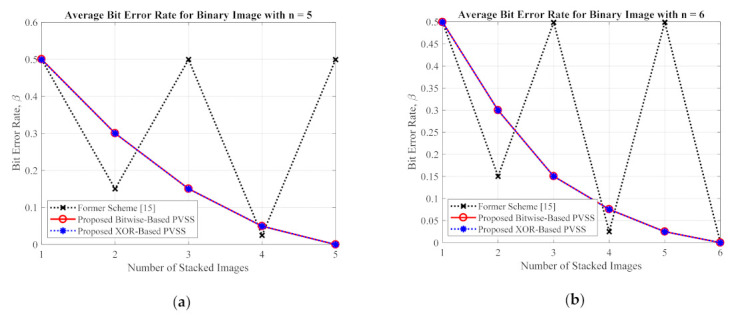
The average bit error rate between the proposed method and the former scheme [15] for a binary secret image with: (**a**) n=5, and (**b**) n=6.

This subsection compares the performances of the proposed method and the former scheme [15] in detail based on an objective image quality assessment. For a binary image, the performance is simply measured and compared under two objective measurements (i.e., an average image contrast and average bit error rate). Herein, four secret images (as shown in Figure 5) were first converted into a set of shared images. The recovery process was subsequently conducted on these generated shared images to produce the recovered secret image. The averages of α and β were then computed for all recovered secret images. Figure 17 and Figure 18 display the performance comparisons in terms of average α and average β, respectively, between the proposed method (with a bitwise and XOR-based approach) and the former scheme [15]. In this experiment, we set the number of shared images as n=5 and n=6. As shown in Figure 17 and Figure 18, the former scheme [15] yields an unacceptable average α and β, respectively, if n or T is an odd number. However, the proposed method performs well for n or T whether they are odd or even numbers. In addition, the proposed method yields progressive results, indicating the increasing average value of α and the decreasing average value of β over different values of n or T.

**Figure 19:**
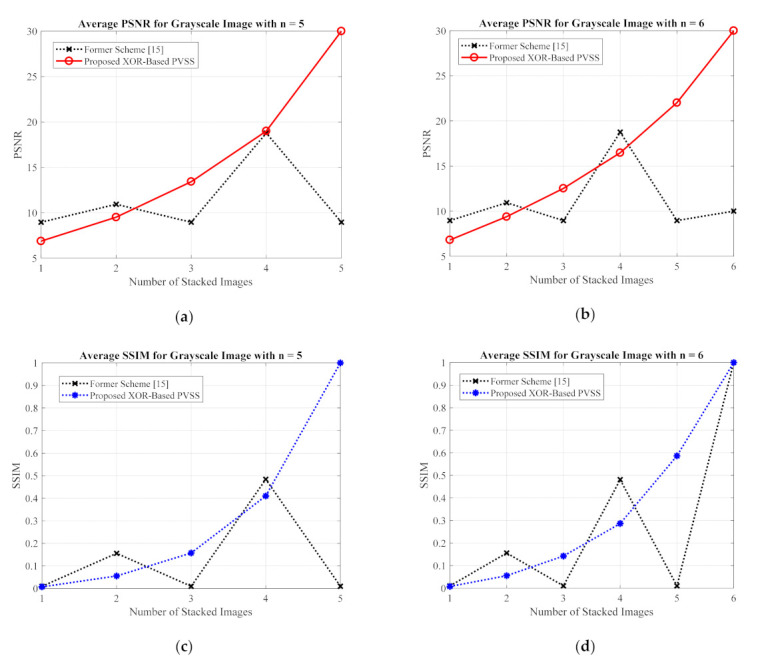

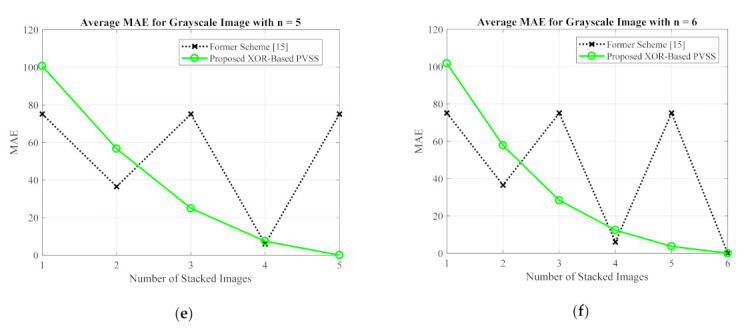
Comparisons between the proposed method and the former scheme [15] in terms of (**a**,**b**) PSNR, (**c**,**d**) SSIM, and (**e**,**f**) MAE values. The comparisons are conducted for a secret image in grayscale format.

**Figure 20:**
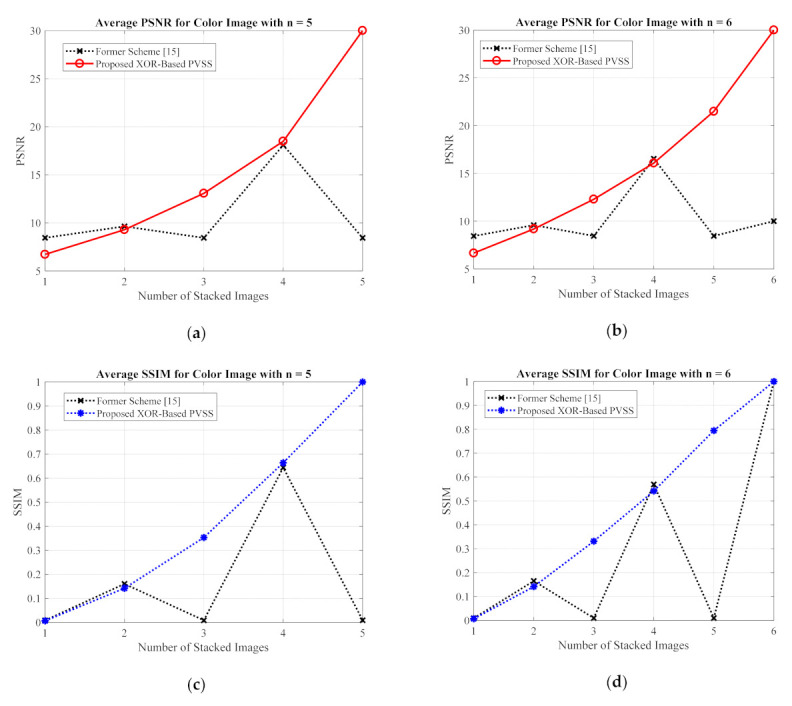

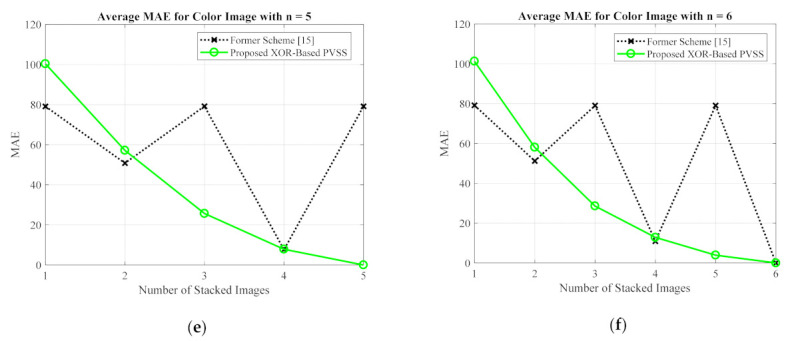
Comparisons between the proposed method and former scheme [15] in terms of (**a**,**b**) PSNR, (**c**,**d**) SSIM, and (**e**,**f**) MAE values. Herein, the secret image is in a color format.

For the grayscale and color images, the comparisons between the proposed method and the former scheme [15] are examined based on the average values of PSNR, SSIM, and MAE. We selected all secret images in grayscale and color spaces shown in Figure 9 and Figure 13 as secret images. All secret images were then converted into a set of shared images. The recovered secret images were further computed by stacking several shared images using an XOR operation. The qualities of all of the recovered secret images were then measured in terms of average PSNR, SSIM, and MAE. Figure 19 and Figure 20 display the performance comparisons for grayscale and color image, respectively. As depicted in these two figures, the former scheme [15] delivers unacceptable results if n or T is an odd number. The proposed method gives correct results whether n or T is an odd or an even number. The proposed method satisfies the progressive constraint for the PVSS task, as indicated by the improving PSNR and SSIM scores that result if more stacked images are utilized in the secret image reconstruction stage. It also gives a good result, decreasing the average MAE value if more stacked images are used to recover a secret image. However, the proposed method is a good candidate for implementing PVSS with adaptive priority and a perfect reconstruction process.

### 4.6. Comparison of Algorithm Aspects for the Proposed Method and Other Schemes

The proposed method and former scheme [15] works on a pixel-by-pixel basis in the shared image generation and secret image reconstruction processes. The computational times of these two methods completely depends on the image size. Let M and N be the width and height of an original secret image. The computational complexity for generating one shared image is $O\left(MN\right)$ for both the proposed method and the former scheme [15]. In reality, the former scheme requires a slightly higher computational burden, since it involves more steps to be conducted in order to compute the shared image, compared to those required in the proposed method. However, the difference is not quite significant. The proposed method and the former scheme [15] need identical computational complexity in the secret image reconstruction process (i.e., $O\left(MN\right)$). These two methods simply perform a stacking process with an XOR operation in order to reconstruct a secret image. However, the proposed method and the former scheme have am almost identical computational complexity, except in terms of the quality of the recovered secret image. Thus, the proposed method is a better choice for implementing a PVSS algorithm.

This subsection also reports the algorithm aspects between the proposed method and other competing schemes. Herein, we simply compared the proposed method with others PVSS schemes [9,10,11,12,13,14,15] based on the share style, encoding matrix, pixel expansion, and adaptive priority weight. Table 1 summarizes this comparison. This table shows that the proposed method is able to perform the PVSS task with an priority adaptive weight similar to that of [12,14,15]. The other schemes cannot utilize the priority adaptive weight in the recovery stage of the secret image. The former approach [15] is the most competitive candidate when compared to the proposed method in these terms. However, the proposed method reveals its superiority since it works regardless of whether the number of stacked shared images is odd or even. The former scheme [15] has a limitation when the number of stacked images is odd. In addition, the proposed method does not require the encoding matrix and pixel expansion in the secret image recovery step, meaning that it requires a lower amount of storage space. In addition, the proposed method generates a set of shared images in the form of a noise-like appearance. Thus, the content of the shared images cannot be easily distinguished from one to the other. The contents of each shared image cannot be easily recognized and perceived by human vision. At the end, the proposed method offers its benefit for the PVSS task with adaptive priority and a perfect reconstruction process for recovering a secret image.

## 5. Conclusions

A simple approach for overcoming the limitation of the former PVSS with adaptive priority weight is presented in this paper. The proposed method is designed to satisfy the lossless constraint and adaptive priority weight required for the PVSS system. The proposed method exploits the bitwise-based and XOR-based techniques for generating a set of shared images. It achieves perfect reconstruction on a recovered secret image whether the number of stacked or collected images is odd or even. While this works for a binary image, the proposed method also works well for grayscale and color images. This superiority can be further applied and extended for video processing or other image processing applications. Thus, the proposed method can be considered and viewed as a strong PVSS alternative with a perfect reconstruction ability.

## Figures and Tables

**Table 1:** Comparisons between the proposed method and the former scheme in terms of algorithm aspects.

Method	Share Style	Encoding Matrix	Pixel Expansion	Adaptive Priority	Quality
Fang’s Scheme [9]	Noise-Like Form	Require	Need	No	Lossless for n is even
Wang’s Scheme [10]	Noise-Like Form	Require	Need	No	-
Hou’s Scheme [11]	Noise-Like Form	Require	No	No	-
Hou’s Scheme [12]	Noise-Like Form	Require	No	Adaptive Priority	Lossy
Lin’s Scheme [13]	Friendly Appearance	No	No	No	Lossy
Yang’s Scheme [14]	Noise-Like Form	Require	No	Adaptive Priority	Lossy
Former Scheme [15]	Noise-Like Form	No	No	Adaptive Priority	Lossy, if n is oddLossless, if n is even
Prasetyo’s Scheme [16]	Noise-Like Form	No	No	No	Lossless for n is odd or even
Proposed Method	Noise-Like Form	No	No	Adaptive Priority	Lossless for n is odd or even

## Data Availability

Not applicable.

## References

[1] Zarepour-Ahmadabadi J., Shiri-Ahmadabadi M., Latif A. (2018). A cellular automata-based multi-stage secret image sharing scheme. Multimed. Tools Appl..

[2] Bharti S.S., Gupta M., Agarwal S. (2018). A novel approach for verifiable (n, n) audio secret sharing scheme. Multimed. Tools Appl..

[3] Liu Y.-N., Zhong Q., Xie M., Chen Z.-B. (2017). A novel multiple-level secret image sharing scheme. Multimed. Tools Appl..

[4] Guo J.-M., Riyono D., Prasetyo H. (2019). Hyperchaos permutation on false-positive-free SVD-based image watermarking. Multimed. Tools Appl..

[5] Prasetyo H., Hsia C.-H. (2019). Improved multiple secret sharing using generalized chaotic image scrambling. Multimed. Tools Appl..

[6] Guo J.M., Riyono D., Prasetyo H. (2018). Improved Beta Chaotic Image Encryption for Multiple Secret Sharing. IEEE Access.

[7] Prasetyo H., Guo J.-M. (2019). A Note on Multiple Secret Sharing Using Chinese Remainder Theorem and Exclusive-OR. IEEE Access.

[8] Yan X., Lu Y. (2017). Contrast-improved visual secret sharing based on random grid for general access structure. Digit. Signal Process..

[9] Fang W.-P., Lin J.-C. (2006). Progressive viewing and sharing of sensitive images. Pattern Recognit. Image Anal..

[10] Wang R.-Z. (2009). Region Incrementing Visual Cryptography. IEEE Signal Process. Lett..

[11] Hou Y.-C., Quan Z.-Y. (2011). Progressive Visual Cryptography with Unexpanded Shares. IEEE Trans. Circuits Syst. Video Technol..

[12] Hou Y.-C., Quan Z.-Y., Tsai C.-F. (2015). A privilege-based visual secret sharing model. J. Vis. Commun. Image Represent..

[13] Lin C.-H., Lee Y.-S., Chen T.-H. (2015). Friendly progressive random-grid-based visual secret sharing with adaptive contrast. J. Vis. Commun. Image Represent..

[14] Yang C.-N., Liao J.-K., Wang D.-S. (2017). New privilege-based visual cryptography with arbitrary privilege levels. J. Vis. Commun. Image Represent..

[15] Chao H.-C., Fan T.-Y. (2017). Random-grid based progressive visual secret sharing scheme with adaptive priority. Digit. Signal Process..

[16] Prasetyo H., Hsia C.-H. (2019). Lossless progressive secret sharing for grayscale and color images. Multimed. Tools Appl..

